# Long non-coding RNA HOTAIR regulates cyclin J via inhibition of microRNA-205 expression in bladder cancer

**DOI:** 10.1038/cddis.2015.269

**Published:** 2015-10-15

**Authors:** X Sun, P Du, W Yuan, Z Du, M Yu, X Yu, T Hu

**Affiliations:** 1 Department of Immunology, School of Basic Medical Science, Binzhou Medical University, Yantai, Shandong, China; 2 Experimental Teaching Center, Binzhou Medical University, Yantai, Shandong, China

## Abstract

The level of microRNA-205 (miR-205) is commonly deregulated in a number of cancers. Through the screening of the microRNA expression profile in bladder cancer tissue and cell lines, we found that expression of miR-205 was significantly suppressed. In addition, the levels of miR-205 expression had a negative correlation with the degree of bladder cancer malignancy. However, the biological functions of miR-205 remained unclear. In this study, we have demonstrated that miR-205 had a role in the inhibition of proliferation, migration and invasion of bladder cancer cells. Moreover, we have identified cyclin J (*CCNJ)* gene, which is involved in cell cycle regulation, as a novel target for miR-205. Furthermore, a long non-coding RNA HOTAIR (HOX transcript antisense RNA) was observed to participate in the silencing of miR-205 in bladder cancer cells by breaking the balance of histone modification between H3K4me3 (histone H3 at lysine 4 methylation) and H3K27me3 on miR-205 promoter. This study elucidates an important role that miR-205 had in the regulation of proliferation, migration and invasion of bladder cancer cells, suggesting a potential therapeutic target for combating bladder cancer.

Bladder cancer is the 7th most common cancer in the world, and ranks fourth among males in economically developed countries.^1^ Although about 70–75% of the cases are nonmuscle-invasive disease based on the initial diagnosis, the local recurrence rate remains high, at ~70%. Moreover, about one fifth of the cases could progress to muscle-invasive bladder cancer, which has a strong tendency toward deadly metastasis.^2^ Understanding the mechanisms underlying the progression of bladder cancer could not only help us find potential novel approaches or agents that inhibit the invasion of tumors, but also increase the therapeutic efficiency on bladder cancer.

MicroRNAs (miRNAs) are a group of small, single stranded, non-coding RNAs that post-transcriptionally downregulate the expression of many genes. The changes in the levels of miRNA expression have been observed in many human malignancies, and affected numerous pathways involved in apoptosis, proliferation, survival and invasion.^3^ Earlier studies have shown that profiling of miRNA expression can be used as a tumor marker and a prognostic tool to predict patient outcome.^4, 5^ In bladder cancers, dozens of miRNAs have showed aberrant expressions including miR-205, which was significantly downregulated in cancerous tissue. Wszolek *et al.*^6^ have reported that reduced expression of miR-200 families (miR-200a, miR-200b, miR-200c and miR-205) in invasive urothelial carcinoma of the bladder; Wang *et al.*^7^ found that the urinary miR-200 family levels were depressed in patients with bladder cancer, and suggested levels of these miRNAs in urine could be developed as noninvasive markers for bladder cancer; a high ratio due to high expression of miR-21 and low expression of miR-205 has been identified as the marker to distinguish between invasive and noninvasive bladder tumors with high sensitivity and specificity.^8^ However, most of these research only revealed the expression pattern of microRNAs, but downstream functions of microRNAs remain to be elucidated, and the characterization of the target genes of microRNA is essential for in-depth understanding of cancer progression.

Also in bladder cancer, cyclin J (CCNJ), a member of the cyclin family, has been detected to be aberrantly expressed. CCNJ is a protein that controls cell mitosis, and a unique cyclin with exclusive maternal expression pattern, suggesting its ability to regulate oogenesis and embryogenesis.^9, 10^ The potential oncogenic role of CCNJ in bladder tumorigenesis has yet to be explored.

Long non-coding RNAs (lncRNA) represent another class of non-coding RNA, which have emerged as a new player in gene regulations, including carcinogenesis and metastasis.^11^ One of the lncRNA, HOX transcript antisense RNA (HOTAIR), have shown to repress gene expression through recruitment of chromatin modifiers.^12^ Although the 5′ end of HOTAIR binds to polycomb repressive complex 2 (PRC2), its 3′ end binds to lysine (K)-specific demethylase 1A (LSD1) complex, giving HOTAIR the abilities to bind to a histone methylase and demethylase. Therefore, HOTAIR can serve as a scaffold for multiple histone modification complexes. This bifunctional character of HOTAIR could be required for coordinating histone modifications of H3K27 methylation and H3K4 demethylation for epigenetic gene silencing in the metastatic processes.^13, 14^ In addition, recent studies have shown that HOTAIR could modulate other classes of non-coding RNAs, including miRNAs,^15^ for example, Wiklund *et al.*^16^ study reported that miR-200 and miR-205 were silenced in bladder cancer, and DNA hypermethylation as possible prognostic markers, but the interaction between HOTAIR and miR-205 has not been reported before.

In this study, we first examined the expression profile of miRNAs in bladder cancer tissues, and found that miR-205 was the most downregulated. Mechanistic studies using bladder cancer cells T24 overexpressed with miR-205 further revealed the modulation of *CCNJ* expression by miR-205. As the expression of lncRNA HOTAIR was upregulated in bladder cancer, we speculate that HOTAIR could mediate the silencing of miR-205 through its regulation on the histone modification. Therefore, this study will shed some light on the role of miR-205 in the progression and prognosis of bladder cancer, and helping determine if miR-205 could be used as a potential target for the treatment of bladder cancer.

## Results

### The expression profile of microRNAs in bladder cancer

**Figure 1:**
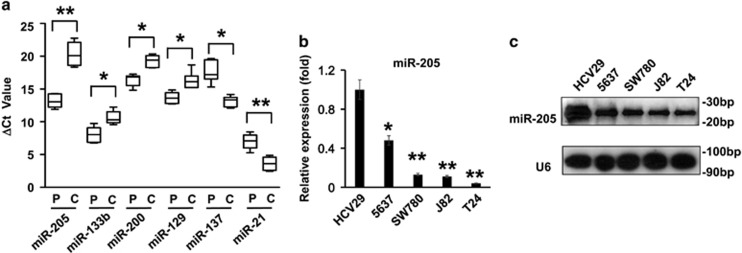
Expression profile of microRNAs in bladder cancer. (**a**) Expression levels of miRNAs in bladder cancer samples. Primary specimens collected from patients with bladder cancer were screened by miRNA-PCR array. The relative expressions of miR-205, -133b, -200, -129, -137 and -21 in bladder cancer tissue (C) and paracarcinoma (P) are shown. (**b**) Relative expression level of miR-205 in different bladder cancer cell lines were analyzed by qRT-PCR. Normal urinary tract cells HCV29 served as control. (**c**) Representative image of a northern blot showing the expression levels of miR-205 in bladder cancer cell lines. **P<0.001 and *P<0.05 compared with the control. Data represent the results from three independent experiments

We firstly selected six microRNAs, the miR-205, miR-133b, miR-200, miR-129, miR-137 and miR-21 that have been revealed to be correlated with bladder cancer.^6, 7, 17^ To investigate the expression levels of these microRNAs in our study, we collected clinical specimens from patients with bladder cancer, and analyzed changes in the expression profile of these miRNAs using a miRNA-PCR array. Among all the miRNAs that were examined, miR-205 was identified as the most downregulated one by quantitative reverse transcription PCR (qRT-PCR), and level of miR-205 expression was found suppressed about 80% as compared with that in control samples, suggesting a drastic down regulation with the maximum inhibition (*P*<0.001; Figure 1a). The levels of miR-133b, miR-200 and miR-129 were all decreased significantly (*P*<0.05), although their extents were less than miR-205. However, the levels of miR-137 and miR-21 were elevated in the bladder patients in our study (*P*<0.05), which was not consistent with the previous study.^18, 19^

Next, we examined the expression levels of miR-205 in a series of bladder cancer cell lines including 5637, T24, J82 and SW780 with immortalized normal urinary tract cells HCV29 as the control. Both qRT-PCR assay (Figure 1b) and northern blot assay (Figure 1c) showed a high level of miR-205 expression in normal HCV29 cells, and the expression miR-205 was decreased as the degree of malignancy increased. In addition, there was limited expression of miR-205 in T24, J82 and SW780 cells.

### Effect of miR-205 on bladder cancer cells growth and cell cycle

**Figure 2:**
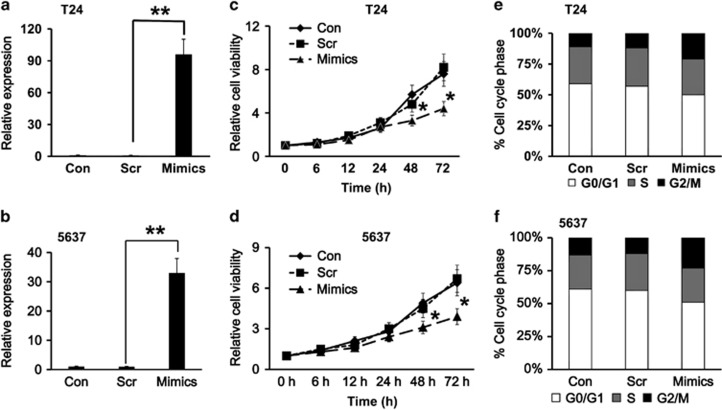
Effect of miR-205 on the growth and cell cycle of bladder cancer cells. The relative expression levels of miR-205 in T24 cells (**a**) and 5637 cells (**b**). Bladder cancer cells T24 or 5637 were transiently transfected with 50 nM of miR-205 mimics (Mimics) or Scramble (Scr). qRT-PCR was conducted, and both wild-type bladder cancer cells (Con) and Scr were used as the negative controls. Proliferation curve in bladder cancer cells T24 (**c**) or 5637 (**d**) as measured by CCK-8 assay. After transfection of mimics or scramble into these cells, the cell proliferation was monitored at various time points. Cell cycle analysis showing percentage of G0/G1, S and G2/M phase in T24 cells (**e**) or 5637 cells (**f**) transfected with mimics or scramble or wild-type cells (Con) as measured by flow cytometry. **P<0.001 and *P<0.05 compared with the negative control. Data represent the results of three independent experiments

To investigate the biological function of miR-205, the gain-of-function experiments were designed. First, miR-205 mimics, which are chemically synthesized fragments with the same sequence as miR-205 and with enhanced miR-205 activity, were transiently transfected into T24 cells and 5637 cells. The overexpression of miR-205 mimics in these bladder cancer cells was confirmed by qRT-PCR with the Scramble (Scr) served as the negative control (Figures 2a and b). CCK-8 assay was employed to study the effect of miR-205 on bladder cancer cells growth. As shown in Figure 2c, T24 cells with the overexpression of miR-205 mimics had a lower proliferation rate compared with that in cells transfected with scramble (*P*<0.05), and cell growth was drastically slowed down after 48 h. Moreover, when the 5637 cells were transfected with miR-205 mimics, this suppression of cell growth also was observed (Figure 2d). Next, both T24 cells and 5637 cells transfected with miR-205 mimics or scramble were analyzed for cell cycle distribution. The result showed that overexpression of miR-205 had a significant suppressive effect on the proliferation in both cells, possibly due to the induction of G2/M cell cycle arrest (Figures 2e and f).

### Effect of miR-205 on bladder cancer cells migration and invasion

**Figure 3:**
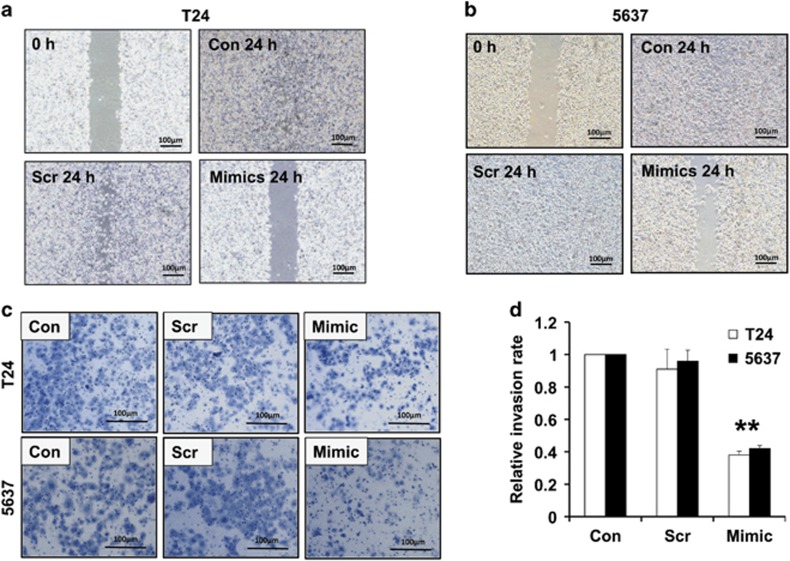
Effect of miR-205 on the migration and invasion of bladder cancer cells. (**a**) Wound healing assay showing the effect of miR-205 on the migration of bladder cancer T24 cells, and (**b**) wound healing assay of bladder cancer 5637 cells. A wound gap was created on the surface of T24 or 5637 cells transfected with miR-205 mimics (Mimics) or scramble (Scr), and the width of gap was measured 24 h after. WT T24 cells (Con) or Scr served as controls. Similarly, invasion assay was conducted in those cells. (**c**) Transwell analysis showing the effects of miR-205 on the invasion of bladder cancer T24 and 5637 cells, and (**d**) showing the quantitative analysis of the invasion ability of T24 and 5637 cells transfected with scramble or miR-205 mimics. **P<0.001 compared with the negative control. Data represent the results of three independent experiments

To study the effect of miR-205 on bladder cancer cell migration, wound healing assay was conducted in T24 cells and 5637 cells that were transiently transfected with miR-205 mimics or scramble, and migration in both cell lines were examined in a series of time points after the wound gaps were created by a p-20 pipet tip scratch. The results showed the wound gap from the wild-type (WT) control cells (Con) or scramble group (Scr) cells of both the T24 and 5637 cells were either fully or nearly recovered 24 h after the established wound gap (Figures 3a and b). However, the gap remained wide in the cells transfected with miR-205 mimic (Mimics). The migration distance of cells with overexpression of miR-205 mimic was significantly wider than that in the cells with scrambles (Figures 3a and b). In addition, transwells were used to examine the invasion capability in those different groups of cells. The results showed the invasion ability of T24 and 5637 bladder cancer cells were dramatically impaired in the presence of overexpressed miR-205 as compared with the scramble or control cells (Figures 3c and d), suggesting that upregulation of miR-205 inhibited the migration and invasion of bladder cancer cells.

### CCNJ is a downstream target of miR-205

**Figure 4:**
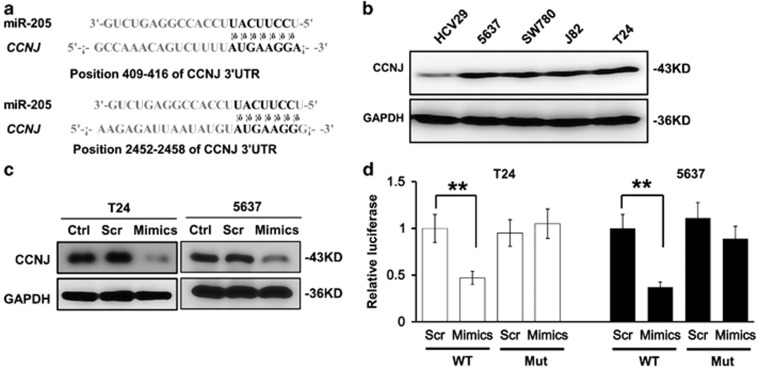
CCNJ is a downstream target for miR-205. (**a**) Putative miR-205-binding sites on *CCNJ* 3′UTR with potential complementary residues shown in black. (**b**) Western blot analysis showing the protein levels of CCNJ in different bladder cancer cell lines as compared with that in normal urinary tract cells HCV29. GAPDH served as a loading control. (**c**) Western blot analysis showing the protein levels of CCNJ in T24 cells transfected with Scramble (Scr), miR-205 mimics (Mimics) and wild-type (WT) cells (Con). GAPDH served as a loading control. The putative binding sites of miR-205 on the CCNJ promoter were mutated. Luciferase vectors (luc *CCNJ* 3′*UTR*) carrying WT or mutated (Mut) sequences of CCNJ 3′UTR were co-transfected with miR-205 into T24 and 5637 cells. (**d**) Luciferase activities of the WT and Mut 3′UTR of CCNJ mRNA were measured in T24 and 5637 cells. The luciferase activity of renilla was used as internal control. **P<0.001 compared with the control. Data represent the results of three independent experiments

To investigate the targeting molecules of miR-205 that involved in the modulation of G2/M cell cycle, the targets of miR-205 were searched using TargetScan (Whitehead Institute for Biomedical Research, Cambridge, MA, USA). CCNJ was found to be downstream of miR-205, and it has been linked to the G2/M cell cycle in prostate cancers.^20^ More interestingly, we have found that there are a couple of putative miR-205 binding sites in the 3′ untranslated region (3′UTR) of CCNJ as shown in Figure 4a. So the levels of CCNJ expression were examined in bladder cancer cell lines 5637, T24, J82 and SW780, and in normal urinary tract HCV29 cells. CCNJ expression was found to be much higher in the cells with high degree of malignancy than that in normal urinary tract cells (Figure 4b). Western blot analysis showed that overexpression of miR-205 in T24 cells attenuated the level of CCNJ expression (Figure 4c). To further investigate the molecular mechanism by which miR-205 regulates CCNJ, vectors carrying both the WT 3′UTR of CCNJ gene mRNA and the potential miRNA-205-binding sites mutated were transfected into the T24 and 5637 cells, and the transcriptional activity was examined using luciferase reporter assay. Although the overexpression of miR-205 significantly inhibited the luciferase activity in the WT CCNJ 3′UTR reporter, there were limited changes in cells with the mutated CCNJ 3′UTR reporter, suggesting a direct interaction between miR-205 and the 3′UTR region of CCNJ (Figure 4d). Hence, miR-205 could downregulate the transcriptional activity of CCNJ, thus impair the mitosis progression.

### Effects of miR-205 on tumor formation *in vivo*

**Figure 5:**
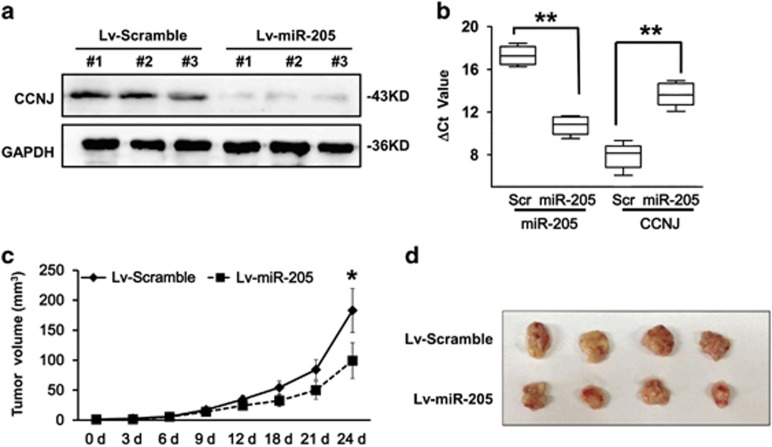
Effects of miR-205 on tumor formation in the xenograft mouse model. (**a**) Western blot showing the levels of CCNJ expression in the subcutaneous tumor tissues isolated from the mice bearing T24 cells with scramble or with miR-205. GAPDH was used as a loading control. (**b**) Relative expression levels of miR-205 and CCNJ in the tumor tissues as measured by qRT-PCR. (**c**) Shown are tumor volume changes in the mice bearing T24 cells with scramble or with miR-205 in a 24-day period. (**d**) Representative images of the tumor formation after 24 days in mice bearing T24 cells with scramble or with miR-205. The experiment was repeated three times. **P<0.001 and *P<0.05 compared with the negative control

A graft mouse model was used to further confirm whether miR-205 could inhibit tumorigenesis *in vivo.* We subcutaneously injected 2 × 10^6^ T24 cells with stable expression of miR-205 (Lv-miR-205) or scramble (Lv-Scramble), respectively, into the flanks of the SCID mice. Tumor sizes were measured, and at the end of time course, tumor tissues were extracted for western blotting and qRT-PCR analysis. Although the western blotting analysis showed a decreased levels of CCNJ expression in tumor-bearing Lv-miR-205 cells (Figure 5a), qRT-PCR analysis confirmed the increased levels of miR-205 expression in tumor-bearing Lv-miR-205 cells than in Lv-miR-205 cells (Figure 5b), suggesting that miR-205 can negatively modulate the expression of CCNJ gene. The initiation and growth of tumor were slower in mice bearing Lv-miR-205 cells than those with Lv-miR-205 cells (Figure 5c), as lower weight and smaller volume of tumors were observed in the mice bearing cells overexpressed with miR-205 (Figure 5d). Thus, our animal experiments confirmed that the expression of miR-205 could inhibit bladder cancer development *in vivo*.

### Histone modification on miR-205 transcription

**Figure 6:**
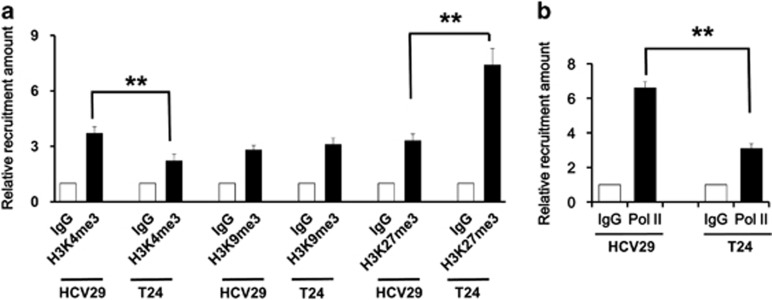
Histone modification on miR-205 transcription. (**a**) ChIP assay showing the recruitment levels of H3K4me3, H3K9me3 and H3K27me3 at the promote region of miR-205 in T24 cells as compared with those in normal urinary tract cells HCV29. (**b**) ChIP assay showing the recruitment levels of polymerase II on the miR-205 promoter in bladder cancer tissues as compared with that in paracarcinoma tissues. The experiment was repeated three times. **P<0.001 compared with the negative control

After our investigations on the downstream target and the function of miR-205, we next explored the mechanisms underlying the silence of miR-205 in bladder cancer. Epigenetic modifications, especially methylation at specific histone sites has an important role in gene expression, and DNA methylation and histone modifications can affect the expressions of miRNAs.^21^ Chromatin immunoprecipitation (ChIP) assay was employed to find a direct correlations between the promoter region of miR-205 and different histone modifications: H3K4me3, H3K9me3 and H3K27me3. The result showed that the levels of H3K4me3 were decreased, and the levels of H3K27me3 were increased significantly in bladder cancer cells T24 compared with those levels in normal HCV29 cells (Figure 6a). As a result, the recruitment of polymerase II was much lower in bladder cancer cells than that in normal cells, an indication that the status of chromosome in miR-205 promoter was much compact in bladder cancer cells than in control cells (Figure 6b). These results implied that the level of miR-205 expression was regulated by the histone methylation status of its promoter.

### Effects of HOTAIR on miR-205 transcription

**Figure 7:**
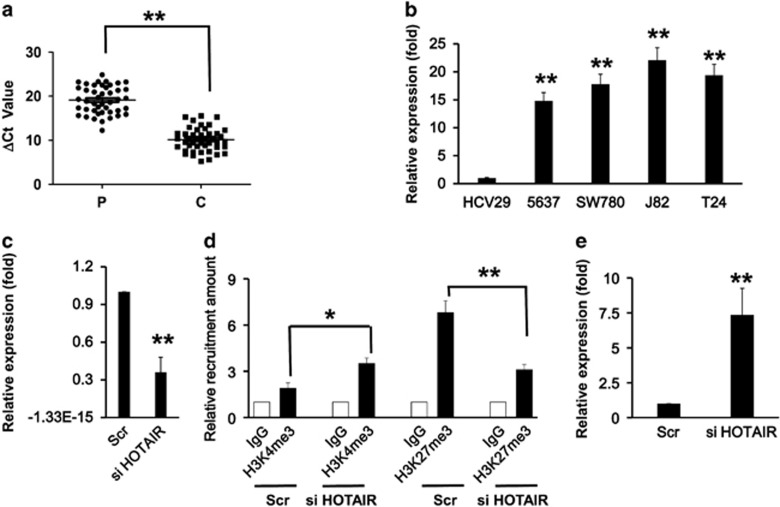
Effects of HOTAIR on the transcriptional level of miR-205. (**a**) qRT-PCR analysis showing the relative expression levels of HOTAIR in primary specimens from patients with bladder cancer. The average levels of HOTAIR expression in bladder cancer tissue (C) and paracarcinoma (P) were compared. (**b**) qRT-PCR analysis showing the relative expression levels of HOTAIR in bladder cancer cell lines and normal urinary tract cells HCV29. (**c**) qRT-PCR analysis showing the levels of HOTAIR in T24 cells with knocking down by specific siRNA HOTAIR (siHOTAIR) or non-target control (Scr). Scr was used as a control. (**d**) ChIP assay showed the histone modification levels of H3K4me3 and H3K27me3 at the promote region of miR-205 with HOTAIR knockdown (siHOTAIR) in T24 cells. Scramble (Scr) was used as the negative control. (**e**) Relative expression levels of miR-205 in T24 cells with knocking down of HOTAIR by siRNA (siHOTAIR). **P<0.001 and *P<0.05 compared with the control. Data represent the results of three independent experiments

Previous studies have shown that HOTAIR binds to both PRC2 that methylates histone H3 at lysine 27 (H3K27) and LSD1 complex that demethylates histone H3 at lysine 4 (H3K4) *in vitro* and *in vivo*.^22^ In addition, our results showed a low H3K4me3 level and a high H3K27me3 level for miR-205 promoter in bladder cancer cells. Therefore, we set to explore if HOTAIR may have a role in the H3K4me3 loss and H3K27me3 gain at miR-205 promoter. Using the RNA samples that were extracted from primary specimens of the patients with bladder cancer, qRT-PCR assay revealed that there were indeed the expressions of HOTAIR in the bladder cancer tissues, and the expression levels were much higher in bladder cancer tissues than that in the paracarcinoma tissues (Figure 7a). *In vitro* study with bladder cancer cell lines yielded consistent results with a 20-fold increase of the HOTAIR expression in bladder cancer cell lines when compared with that in normal HCV29 cells (Figure 7b). When the levels of HOTAIR expression was knocked down in T24 cells using specific small interfering RNA (siRNA; Figure 7c), and the protein levels of PRC2 and LSD1 were not changed (data not shown). Interestingly, the recruitment of H3K4me3 and H3K27me3 on miR-205 promoter was changed significantly, and was almost recovered to the levels in HCV29 cells (Figure 7d). Moreover, the silencing of miR-205 expression in T24 cells with imbalanced H3K4me3 and H3K4me3 was reversed in T24 cells with knockeddown HOTAIR expression (Figure 7e), suggesting a direct role of HOTAIR on the regulation of miR-205 expression via the recruiting PRC2 and LSD1 in bladder cancer.

## Discussion

miRNAs represent a new class of potential therapeutic targets for many complex diseases, especially for the treatment of cancer. Several studies have demonstrated that one of its members, miR-205, can regulate the proliferations of several types of cancer cells: targeting ZEB1 (zinc finger E-box binding) and Ubc13 in breast cancer cells,^23^ ZEB2 in renal cell carcinoma,^24^ phosphatase and tensin homolog in endometrial cancer Ishikawa cells,^25^ Yin Yang 1 in gastric cancer cells,^26^ Ezrin and Lamin A/C in ovarian cancer,^27^ just to name a few. The role of miR-205 is rather complex, since it can act either as an oncogene or a tumor suppressor gene under different cell contexts.^28^ In this paper, we have observed the G2/M arrest in T24 cells with overexpressed miR-205, and identified a mitosis controlling cyclin, CCNJ, which was downregulated by miR-205 via targeting its 3′UTR, as a novel target of miR-205 in bladder cancer.

Our study on the primary specimens from patients with bladder cancer revealed that the levels of miR-205 expression was significantly downregulated in bladder cancer tissues as compared with those in the paracancerous tissues, and this downregulation of miR-205 was correlated with the grades of malignancy. Furthermore, our results showed that increased miR-205 expression could significantly slow down the proliferation, migration and invasion of bladder cancer cells, suggesting that miR-205 behaves like a tumor suppressor gene in bladder cancer. However, Gottardo *et al.*^29^ reported that expression of miR-205 was significantly upregulated in bladder cancers compared with normal bladder mucosa. Ratert *et al.*^30^ found miR-205 in malignant bladder tissue samples compared with healthy tissue, and these results were not consistent with the majority as well as ours. As they discussed, some important limitations of their studies have to be noted: retrospective study, selection bias of samples, the availability of suitable tissue and absence of a set of nonmalignant control samples, all affected miRNA expression differences. Our expression of miR-205 was examined in 44 freshly collected patients, and the expression pattern was consistent with the majority reports. Nevertheless, we only have 44 patients, enlargement of sample number should be considered in further studies. In addition, we have identified CCNJ as a direct target for miR-205. Mechanistically, miR-205 regulates proliferation, migration and invasion via its binding to the 3′UTR of CCNJ mRNA. Previous studies have already categorized CCNJ as a putative oncogene: it was found to be embedded in a cluster of dysregulated genes in pediatric high-risk B-precursor acute lymphoblastic leukemia;^31^ report from Feliciano *et al.*^32^ showed that it was not expressed in normal HMEC cells, but in breast cancer cells MCF7 and MDA-MB-231, and when CCNJ was silenced, the proliferation of MCF7 cells were decreased due to G2/M cell cycle arrest. We found that CCNJ was highly expressed in bladder tissues and bladder cancer cells, indicating that CCNJ may also participate in the bladder tumorigenesis.

Human HOTAIR, a 2.2 kb lncRNA transcribed from the HOXC locus, binds both to the PRC2 and the LSD1 complexes, and it was recruited to hundreds of genomic sites to promote coordinated H3K27 methylation and H3K4 demethylation, respectively.^33, 34^ There was only one report by Yan *et al.*,^35^ showing that HOTAIR is upregulated in bladder cancer, and it can be used as a prognostic marker to predict the recurrence in stage Ta/T1 bladder cancer. However, the understanding on the function of HOTAIR is still scarce in bladder cancer. In this study, we found that the levels of the HOTAIR expression were much higher in bladder tissues and in bladder cancer cells. In addition, HOTAIR could break the balance between H3K4me3 and H3K27me3 on the promoter region of miR-205 hereby blocking the expression of miR-205. To date, there are only limited papers exploring the regulation of a miRNA expression by a lncRNA, especially through modulation of the histone modification on the miRNA promoter. We hope that the novel discovery presented in this paper would shed a bright light on bridging the crosstalk between lncRNAs and miRNAs.

Moreover, we believe that the upregulated expression of miR-205 that we have previously observed in low-grade noninvasive bladder urothelial cancer slows down the proliferation. In the more aggressive forms of urothelial cancers, the expression of miR-205 was silenced, resulting in the increase in cell proliferation and invasion. In summary, our studies on the effect of miRNA-205 in bladder cancer brings new knowledge in bladder tumorgenesis, and further studies on miRNA-205 could establish it as a potential tumor marker and new target for treatment of advanced bladder cancer.

## Materials and Methods

### Primary bladder cancer specimens

Primary specimens from bladder cancer patients were obtained from Department of Oncology, Binzhou Medical College Affiliated Hospital with signed informed consent from patients. We collected 44 patients with bladder cancer from September 2009 to August 2014. To detect the microRNA levels in tumor tissues and para-tumor (defined as >2.0 cm distance from tumor edge) were taken and stored at liquid nitrogen until detection. This study was also approved by the Ethic Committee Board of Binzhou Medical College, Binzhou, Shandong, China.

### Cell cultures

HCV29, 5637, T24, J82 and SW780 cells were purchased from ATCC (Manassas, VA, USA), cultured in high-glucose DMEM containing 10% fetal bovine serum, and incubated at 37 °C in a humidified atmosphere with 5% CO_2_.

### Oligonucleotide transfection, plasmid construction and lentiviral infection

The scramble and miR-205 mimics were obtained from RiboBio (RiboBio Co., Ltd., Guangzhou, China), with scramble served as the negative control. The cells were transfected with those oligos using Lipofectamine 2000 (Life Technologies, Grand Island, NY, USA) according to the manufacturer's protocol, and the culture was replaced with fresh medium 6–8 h after the transfection.

Luc-CCNJ 3′UTR was commercially constructed by RiboBio and the mutations of potential miR-205-binding sites on the Luc-CCNJ 3′UTR plasmid were performed by TransGen (TransGen Biotech Co., Ltd., Beijing, China). siRNA specially targeting EZH2, LSD1 and non-target siRNA control were purchased form Sigma-Aldrich (St. Louis, MO, USA), and siRNA specially targeting HOTAIR was custom synthesized by TransGen with sequence of 5′-GAACGGGAGUACAGAGAGAUU-3′. The prepackaged lentivirus was purchased from GeneChem (Quebec, Canada). Lentivirus carrying scramble or miR-205 was packaged in the HEK-293T cells and collected from the supernatants following the manufacturer's protocol. Stable cell lines were established by infecting the lentivirus into T24 cells using puromycin as a selection marker.

### Quantitative reverse transcription PCR (qRT-PCR)

Total RNAs were isolated using an RNAzol reagent (Life Technology, San Francisco, CA, USA) based on the manufacturer's instructions. Briefly, complementary DNA was reverse transcribed from 1 *μ*g of total RNA using an M-MLV reverse transcriptase (Promega, Madison, WI, USA) in a 25-*μ*l reaction mixture. qRT-PCR was carried out in the ABI 7300 reverse transcription RT-PCR system with the SYBR Green Reverse transcription PCR Master Mix (TOYOBO, Osaka, Japan). The primers used in the reaction for miR-205, miR-133b, miR-200, miR-129, miR-137 and miR-21 were purchased from RiboBio (Guangzhou RiboBio Co., Guangzhou, China). The expression level of glyceraldehyde 3-phosphate dehydrogenase (GAPDH) was served as internal control. In addition, following primers were also used:

*CCNJ*: F 5′-cctgcgcgagaaggaactg-3′ R 5′-cgttgtagcgatccatgaagtg-3′. *HOTAIR*: F 5′-ggtagaaaaagcaaccacgaagc-3′ R 5′-acataaacctctgtctgtgagtgcc-3′. *GAPDH*: F 5′-cgaccactttgtcaagctca-3′ R 5′-aggggtctacatggcaactg-3′.

### Western blotting analysis

Cells were lysed with 1 × radioimmunoprecipitation assay buffer (20 mM Tris-HCl (pH 7.5), 150 mM NaCl, 1 mM Na_2_EDTA, 1 mM EGTA) (Cell Signaling Technology, Danvers, MA, USA), 50 *μ*g of total protein was subjected to 4–12% SDS-polyacrylamide gel electrophoresis (SDS-PAGE) gel (Life Techonology), transferred onto nitrocellulose membranes (BioRad, Hercules, CA, USA), and immunoblotted with 1st antibodies against CCNJ (1 : 200) or GAPDH (1 : 1000) (Santa Cruz, Dallas, TX, USA). After washed with PBS three times for 10 min each, the member was incubated with goat-anti-rabbit 2nd antibody (1 : 2000) for 2 h. The hybrid signal was detected using Pierce ECL Western Blotting Substrate (Life Technology).

### Northern blot analysis

Expression of miR-205 in bladder cell lines were examined by northern blot using a ^32^P-labeled oligo (5′-CAGACTCCGGTGGAATGAAGGA-3′). Briefly, total RNA was extracted from cells using Trizol (Life Technology), 30 *μ*g of total RNA was heated at 70 °C for 5 min and electrophoresized in a 15% denaturing PAGE gel, then transferred to a nylon membrane (BioRad), prehybridized in hybridization buffer (Sigma-Aldrich, Natick, MA, USA) for 1 h at 37 °C, and then hybridized overnight with the ^32^P-labeled oligo at 40 °C. MiR-205-radiolabelled probes were removed from membranes by washing twice with and the hybrid signals are exposed to a high-sensitive X-ray film overnight. An antisense oligo of U6 (5′-AAAATATGGAACGCTTCACGA-3′) was used to detect U6 snRNA from each sample as a loading control.

### Luciferase assays

A total of 10 000 T24 and 5637 cells were seeded in the well of a 24-well plate. When the cells reached 60% confluence, they were co-transfected with WT Luc-CCNJ 3′UTR or mutant Luc-CCNJ 3′UTR plasmids and either scramble or miR-205 mimics, using VigoFect (Vigorous, Beijing, China) according to the manufacturer's instructions. After 48 h, luciferase activity was measured following the manufacturer's instructions and normalized against the Renilla luciferase activity using Dual-Luciferase Reporter Assay System Technical Manual (Promega).

### CCK-8 assay

T24 and 5637 cells were seeded in a 96-well plate at a density of 2000 cells per well. The absorptions of the cells were measured after various incubation time using a CCK-8 kit (Dojindo Laboratories, Kumamoto, Japan) according to the manufacturer's instruction. Absorbance was measured at OD 450 nm after cells were stained and the relative cell proliferation rate was normalized with the value at day 0.

### Flow cytometry analysis

Cells were washed with 1 × PBS, trypsinized, and fixed with 70% ethanol for 30 min on ice. RNA was degraded by incubation with 20 mg/ml RNase (Sigma-Aldrich) at 37 °C for 1 h. Cells were then labeled with 20 mg/ml propidium iodide (Sigma-Aldrich), and DNA content was assessed by fluorescence-activated cell sorting Calibur flow cytometry (Becton Dickinson, Franklin Lakes, NJ, USA) and analyzed by the ModiFit LT v2.0 software (Verity Software House, Inc., Topsham, ME, USA).

### Wound healing assay

When the cells in a six-well plate reached 90% confluence, the wound gaps were generated by using a 20 *μ*l pipet tip to scratch the surface of cell layer. After PBS wash, cells were cultured and monitored. The wounded gaps were photographed at different time points, and analyzed by measuring the distances of migrating cells from five different areas for each wound.

### Cell invasion assay

Invasion assay was performed using the 24-well BD matrigel invasion chambers (BD Biosciences, Cowley, UK) in accordance with the manufacturer's instructions. Briefly, in the upper well of the invasion chamber, 5 × 10^4^ cells were seeded per well in DMEM without serum; in the lower chamber well, DMEM supplemented with 10% FBS were added to stimulate the cell invasion. After 24-h incubation, non-invading cells were removed from the top well with a cotton swab. The migrated cells, which were in the bottom surface, were fixed with 3% paraformaldehyde, stained with 0.1% crystal violet and photographed in three independent fields for each sample. They were further extracted with 33% acetic acid and their absorbance was measured quantitatively using a standard microplate reader at a wavelength of 570 nM.

### ChIP assay

Cells were fixed with 4% formaldehyde and sonicated to prepare the chromatin fragments. Chromatin samples were immunoprecipitated with antibodies against H3K4me3, H3K9me3, H3K27me3, EZH2, LSD1 or normal rabbit IgG antibodies at 4 °C for 3 h. After crosslinking reversal, precipitated DNA was analyzed by PCR to detect a 151 bp fragment from the promoter region of miR-205 using the following primers: forward, 5′-GGATTTAGGGAGGAAGAA-3′ reverse, 5′-ACAAGGAAGAGGCCAGAG-3′. The data were normalized against that of corresponding DNA precipitated by rabbit IgG.

### *In vivo* mouse experiments

The animal experiments were carried out in strict accordance with the recommendations in the Guide for the Care and Use of Laboratory Animals of the National Institutes of Health. Xenograft tumor model was established by subcutaneous injection of 2 × 10^6^ cells into the left and right oxter flank of SCID mice. There were six mice per group, and tumor sizes were measured every 3 days using a vernier caliper when it was visible. The tumor volume was calculated using the formula: volume=0.5 × length × width^2^.

### Statistical analysis

The data are expressed as mean±S.D. Comparisons between groups were analyzed using Student's *t*-test or ANOVA, and the Student–Newman–Kleuss method was used to estimate the level of significance. Differences were considered to be statistically significant when *P*-value <0.05. All results were reproduced in at least three independent experiments.

## Abbreviations

lncRNA: long non-coding RNA
miRNA: microRNA
HOTAIR: HOX transcript antisense RNA
CCNJ: cyclin J
PRC2: polycomb repressive complex 2
LSD1: lysine (K)-specific demethylase 1A
qRT-PCR: quantitative reverse transcription PCR
3′UTR: 3′ untranslated region
siRNA: small interfering RNA
WT: wild type
Mut: mutant
Con: control
Scr: scramble
H3K4me3: histone H3 at lysine 4 methylation
ZEB: zinc finger E-box binding
GAPDH: glyceraldehyde 3-phosphate dehydrogenase
ChIP: chromatin immunoprecipitation
